# Targeting NANOS1 in triple-negative breast cancer: synergistic effects of digoxin and PD-1 inhibitors in modulating the tumor immune microenvironment

**DOI:** 10.3389/fonc.2024.1536406

**Published:** 2025-01-24

**Authors:** Tangyi Wang, Yadian Lei, Jingwei Sun, Li Wang, Yuxin Lin, Zhijing Wu, Shoude Zhang, Chengzhu Cao, Haiyan Wang

**Affiliations:** ^1^ Department of Basic Medical Sciences, Qinghai University Medical College, Xining, Qinghai, China; ^2^ Department of Medical Laboratory, Qinghai Provincial People’s Hospital, Xining, Qinghai, China; ^3^ State Key Laboratory of Plateau Ecology and Agriculture, Qinghai University, Xining, Qinghai, China; ^4^ Research Center for High Altitude Medicine, Qinghai University, Xining, Qinghai, China; ^5^ Key Laboratory of the Ministry of High Altitude Medicine, Qinghai University, Xining, Qinghai, China

**Keywords:** immune checkpoint blockade, triple-negative breast cancer, malignant phenotype, nanos1, PD-1 inhibitors, tumor microenvironments

## Abstract

**Introduction:**

Triple-negative breast cancer (TNBC) is an aggressive subtype of breast cancer resistant to endocrine and targeted therapies. Immune checkpoint inhibitors (ICIs) have shown significant efficacy in various cancers. *Taraxacum officinale*, commonly known as dandelion, has traditionally been used to treat breast-related diseases and is recognized for its beneficial composition and low side effects. FDA-approved drugs, having undergone rigorous validation for their safety, efficacy, and quality, provide a foundation for drug repurposing research. Researchers may explore FDA-approved drugs targeting the potential target NANOS1 for TOE (*Taraxacum officinal*e extract) treatment to develop innovative therapeutic strategies. In this context, Dig (Digoxin) and AA (Algestone acetophenide) have been identified as potential drug candidates for further exploration of their therapeutic effects and application potential in targeting NANOS1.

**Methods:**

RNA sequencing (RNA-seq) was employed to identify potential targets for triple-negative breast cancer (TNBC) from TOE. Bioinformatics tools, including bc-GenExMiner v4.8, the Human Protein Atlas, and the TIMER database, were utilized for target identification. Molecular docking studies assessed FDA-approved drugs interacting with these targets, with Dig and AA selected as candidate drugs. The therapeutic efficacy of Dig and AA in combination with PD-1 inhibitors was evaluated using the 4T1 mouse model. Flow cytometry was applied to assess lymphocyte infiltration in the tumor immune microenvironment. RNA-seq analysis after target silencing by small interfering RNA (siRNA) was performed, followed by Gene Ontology (GO) and Kyoto Encyclopedia of Genes and Genomes (KEGG) pathway analysis. Validation of findings was conducted through quantitative PCR and Western blot analysis.

**Results:**

TOE inhibited TNBC cell growth, migration, and invasion, as assessed by CCK-8 and transwell assays. RNA-seq indicated the effects may be due to *NANOS1* down-regulation. Survival analysis showed lower *NANOS1* expression correlated with better prognosis. Immunoinfiltration analysis indicated a negative correlation between *NANOS1* levels and activated NK cells. Molecular docking identified Dig and AA as high-affinity binders of *NANOS1*. Animal experiments showed Dig and PD-1 inhibitor combination enhanced immunotherapy efficacy for TNBC.

**Discussion:**

The findings from this study suggest that TOE may offer a novel therapeutic approach for TNBC by targeting *NANOS1*, a protein whose down-regulation is associated with improved patient outcomes. The negative correlation between *NANOS1* and activated NK cells highlights the potential role of the immune system in TNBC pathogenesis and response to treatment. The identification of Dig as potential drugs targeting *NANOS1* provides a new direction for drug repurposing in TNBC. The synergistic effect of Dig and PD-1 inhibition observed in animal models is promising and warrants further investigation into the role of immunotherapy in TNBC treatment. Overall, this study identifies *NANOS1* as a new target for TNBC therapy and suggests a combination therapy approach that could enhance immunotherapy effectiveness and improve patient outcomes.

## 1 Introduction

Breast cancer is among the most prevalent and aggressive malignancies globally. In women, breast cancer remains the leading cause of both morbidity and mortality according to the latest data, with triple-negative breast cancer (TNBC) being one of the most challenging subtypes to treat (1). TNBC is characterized by the absence of progesterone receptors (PR), estrogen receptors (ER), and human epidermal growth factor receptor 2 (HER-2), rendering it unresponsive to endocrine and targeted molecular therapies (2). As a result, chemotherapy continues to be the mainstay of treatment for TNBC, despite its associated adverse effects and long-term regimens, which significantly impair patients’ quality of life (3).

These limitations highlight the urgent need for alternative therapeutic strategies for TNBC. Traditional Chinese medicine (TCM), with its multi-component, multi-target, and synergistic effects, offers promising advantages, including fewer adverse effects compared to conventional treatments. In recent years, the exploration of natural active ingredients from TCM has become a prominent research focus, with many compounds showing promise in cancer treatment (4–8). Among these, *Taraxacum officinale*, commonly known as dandelion, has attracted attention as a traditional herbal remedy with promising potential in breast cancer treatment. Studies have shown that extracts from *T. mongolicum* and *T. formosanum* induce apoptosis in breast cancer cells, reduce cell proliferation, disrupt mitochondrial membrane potential, and affect cell migration *in vitro* (9). *In vivo*, *T. mongolicum* administration in breast cancer-bearing mice has been shown to reduce tumor volume and weight, further supporting its potential as an effective therapeutic agent in breast cancer treatment (10).

Despite these promising findings, the molecular mechanisms underlying TOE’s therapeutic effects remain poorly understood. Previous studies have highlighted the interaction between NANOS1 and PUMILIO2 proteins in human germ cells, suggesting that their interplay plays a significant role in controlling mRNA stability and translation during germ cell development (11). Recent studies have suggested that NANOS1 expression, a key factor in epithelial-mesenchymal transition (EMT), plays a pivotal role in the invasiveness, migratory potential, and stem cell-like properties of breast cancer cells (12). Thus, NANOS1 is not only a potential regulatory factor in the onset and progression of breast cancer but also represents a novel therapeutic target.

Considering the limitations of current therapies for TNBC and the emerging therapeutic potential of targeting NANOS1, this study investigates the repurposing of FDA-approved drugs, specifically Dig and AA, for synergistic use with PD-1 inhibitors. Digoxin, a cardiac glycoside, is widely used for managing heart failure and arrhythmias due to its inhibition of Na+/K+-ATPase, which increases intracellular calcium levels (13). Beyond its cardiovascular applications, digoxin has demonstrated anticancer properties by inducing apoptosis, reducing tumor cell proliferation, and modulating the tumor microenvironment (14–16). These effects are linked to the role of Na+/K+-ATPase in cancer cell signaling and survival (17). Algestone acetophenide, a synthetic progestin commonly used for hormone regulation and anti-inflammatory therapy, exhibits molecular activity by binding to progesterone receptors and modulating downstream pathways, which may suppress inflammatory responses and inhibit cancer cell migration and proliferation (18, 19). The distinct mechanisms of these drugs make them promising candidates for repurposing in TNBC, particularly in targeting NANOS1 and enhancing the efficacy of immune checkpoint inhibitors. This study employed MDA-MB-231 human breast cancer cells and 4T1 murine breast cancer cells as *in vitro* models to investigate the effects of *Taraxacum officinale* extract (TOE) on triple-negative breast cancer (TNBC), focusing on its potential to suppress tumor growth and invasiveness. The findings identified NANOS1 as a potential prognostic marker for breast cancer. Furthermore, the potential of digoxin and algestone acetophenide to modulate NANOS1-mediated pathways was investigated, providing a novel approach for targeting TNBC pathways regulated by NANOS1. By combining these FDA-approved drugs with PD-1 inhibitors, this study seeks to advance understanding of combination therapies for TNBC, thereby addressing the critical need for more effective treatment options.

## 2 Materials and methods

### 2.1 Preparation of TOE

*Taraxacum officinale* (Product Standard No. GHT1091), was purchased from Liaoning Senkangyuan Ecological Agriculture Co. The plant material was refluxed with 75% ethanol for 3 hours at 60°C, followed by filtration and concentration. This process was repeated three times, and the combined extract was purified using a D-101 macroporous resin column, where sugars were removed by water elution. The ethanol leaching fraction was collected, evaporated, and spray-dried to obtain *Taraxacum officinale* extract (TOE).

### 2.2 Chemicals

PD-1inhibitor (PD-1) and Digoxin (Dig) were purchased from Selleckchem. Algestone acetophenide (AA) was purchased from TOPSCIENCE. All chemicals were dissolved in DMSO for the *in vivo* and *in vitro* studies.

### 2.3 Animal model construction

Dissolve 10 g of tribromoethanol (Sigma) in 10 mL of tert-amyl alcohol (Sigma) at room temperature to prepare a crystal-free stock solution. Filter the stock solution using a 0.22 μm filter (Millex-GP SLGPR33RB, Millipore), then dilute it with physiological saline (National Drug Approval No. H11021190; 0.9 g/100 mL) to prepare a 19.2 mg/mL (2.5% avertin) working solution. Incubate the working solution at 37°C for 4 hours, mix thoroughly by shaking, and store at 4°C protected from light. To establish a mammary fat pad tumor model in mice, intraperitoneally inject 2.5% avertin at a dose of 160 μL/10 g body weight (307 mg/kg) for anesthesia. Once fully anesthetized, the mice were placed in a supine position on a sterile workbench, and their skin was disinfected with povidone-iodine. Make a small incision (~0.5 cm) above the fourth right mammary gland using ophthalmic scissors. Carefully lift the skin with a cotton swab to expose the mammary fat pad, then inject 40 μL of serum-free suspension containing 5 × 10^5 4T1 cells into the fat pad. Mice in the monotherapy groups received intraperitoneal injections of Dig (5 mg/kg) daily or AA (10 mg/kg) every two days, and the combination group also received a 5 mg/kg PD-1 inhibitor every two days for 14 days. Tumor volume was measured using calipers, and the formula used was: volume (mm^3) = [width^2 (mm^2) × length (mm)]/2. On the 15th day of treatment, the mice were euthanized with 2.5% avertin anesthesia. Freshly isolated tumors were rapidly dissociated to obtain viable single cells. Tumor-infiltrating cells were analyzed by fluorescence-activated cell sorting (FACS) to assess antitumor immune responses.

### 2.4 Cell culture

The 4T1 and MDA-MB-231 cell lines were obtained from the Procell Life Science & Technology Co., Ltd. MDA-MB-231 cells were cultured in DMEM supplemented with 10% fetal bovine serum and 1% penicillin/streptomycin (Procell). 4T1 cells were cultured in RPMI-1640 supplemented with 10% fetal bovine serum and 1% penicillin/streptomycin (Procell). Cells were cultured at 37°C with a 5% CO2 atmosphere.

### 2.5 CCK8 assays

MDA-MB-231 or 4T1 cells were seeded in a 96‐well plate at 2.0×10^5 cells/mL and cultured in medium at 37°C with a 5% CO2 atmosphere for 24 h. After that, the cells were pretreated with various concentrations of TOE for 24 h. At the end of the stimulation, the medium was removed, and 20 µL of CCK-8 reagent (Elabscience, China) was added to each well, and the incubation was continued for 1 h. The absorbance of each well was measured at 450 nm.

### 2.6 Transwell assay

The migration and invasion abilities of cells were assessed using Transwell chambers (Corning). For invasion assays, 30 µg of Matrigel matrix (Corning) was added to the upper chamber and incubated at 37°C for 1 hour. For migration assays, no Matrigel was added. A total of 200 μL of cells (2.5×10^5/mL, treated with TOE, Dig, or AA at IC50 concentration in serum-free medium) was added to the upper chamber, and 750 μL of serum-containing medium was added to the lower chamber. The cells were incubated for 20 hours at 37°C in a 5% CO2 atmosphere. After incubation, the medium was removed, and the chambers were washed twice with PBS. The cells were fixed with 3.7% formaldehyde in PBS for 15 minutes, washed twice with PBS, stained with Giemsa dye for 10 minutes, and the remaining cells on the upper surface of the membrane were gently wiped away using a cotton swab. The number of migrated cells was then counted.

### 2.7 Transcriptome sequencing

MDA-MB-231 cells were seeded in 10 cm dishes and grown to 90% confluency. 9 mL of complete media or TOE (at a concentration of IC50) were added to the control and treatment groups, respectively. After incubated at 37°C with a 5% CO2 atmosphere for 24 h, the medium was removed and washed twice with PBS. Cells were collected in Trizol (Invitrogen). RNA quality control, library construction, and sequencing were performed by Biomarker Technologies.

MDA-MB-231 cells were seeded into 10 cm culture dishes and incubated until they reached 90% confluence. The knockdown efficiency of siNANOS1#1 was validated by qPCR. The control and treatment groups were transfected with either siNC or siNANOS1#1, respectively, using Lipofectamine 3000 (Thermo Fisher). The cells were incubated at 37°C in a 5% CO2 atmosphere for 48 hours, after which the medium was removed, and the cells were washed twice with PBS. Cells were harvested in TRIzol (Invitrogen). RNA quality control, library construction, and sequencing were performed by GeneWiz Inc.

### 2.8 *NANOS1* expression in breast cancer

Gene expression data were analyzed using the bc-GenExMiner v4.8 platform (http://bcgenex.ico.unicancer.fr/) (20), which integrates publicly available breast cancer transcriptomic data, including 11,359 DNA microarray samples and 4,421 RNA-seq samples. These datasets include healthy breast tissue, adjacent normal tissue, tumor tissue, and various subtypes of breast cancer. The expression of the NANOS1 gene was compared across these tissue types, including healthy tissue, tumor tissue, and triple-negative breast cancer (TNBC) subtypes. Statistical analysis was performed using t-tests or ANOVA to assess the significance of gene expression differences, with a p-value of less than 0.05 considered statistically significant. Gene expression variations were visualized using box plots to provide a clearer representation of differences between groups. Additionally, the platform enables correlation analysis between gene expression data and clinical characteristics, further exploring potential associations between gene expression and clinical outcomes in breast cancer.

### 2.9 Survival analysis

The Human Protein Atlas (THPA, https://www.proteinatlas.org/) (21) provided protein expression from the Tissue Atlas and Pathology Atlas. This platform combines gene expression, protein data, and clinical outcomes to investigate the relationship between NANOS1 expression levels and prognosis in breast cancer patients. The relationship between the *NANOS1* gene and the prognosis of breast cancer patients was analyzed using the Kaplan-Meier Plotter online analysis tool (https://kmplot.com/analysis/) (22) which sources its databases from GEO, EGA, and TCGA. Kaplan-Meier survival analysis was performed to classify patients into high-expression and low-expression groups based on NANOS1 protein levels. Survival curves were generated and compared to evaluate the association between NANOS1 expression and patient survival. The log-rank test was used to assess statistical significance, with a p-value of less than 0.05 considered statistically significant. Through this analysis, The Human Protein Atlas provides valuable insights into the potential of NANOS1 as a prognostic biomarker in breast cancer.

### 2.10 Assessment of immune cell infiltration in TNBC

TIMER2.0 (http://timer.cistrome.org/) was used to assess immune cell infiltration in various cancer types based on deconvolution algorithms (23). The platform allows for the evaluation of the infiltration levels of different immune cells in the tumor microenvironment and correlates these with gene expression and clinical data. In this study, we analyzed the correlation between the expression of the NANOS1 gene and immune cell infiltration in tumor samples from The Cancer Genome Atlas (TCGA). Additionally, TIMER2.0 was used to explore the relationship between immune infiltration and clinical outcomes, such as patient survival and tumor stage. The tool supports analysis across multiple cancer types, providing insights into immune response patterns and the potential role of NANOS1 in immune evasion and immunotherapy.

### 2.11 High-throughput virtual molecular docking screening

The structure of NANOS1 protein was obtained from the PDB database with PDB number 4CQO (24). Hydrogen atoms were added using UCSF Chimera software and protonated states were assigned using the H++3.0 program (25). SiteMap (26) was then used to predict the optimal small molecule binding site.

AutoDock Vina1.2.0 software (27) was used for high-throughput virtual screening, and the active site predicted by SiteMap was set as a docking center with docking center coordinates X,Y and Z of 14.25, -5.6, and 50.11, respectively, and the box size was set as a square with a side length of 22.5 Å. The conformation is sampled and scored using a genetic algorithm, and the best conformation is selected by ranking the conformations according to the docking score.

### 2.12 Virtual screening

3177 molecules from the ZINC database were used as screening targets in this study, and they are all drug molecules approved for various applications. Some potential drug molecules that may bind to the NANOS1 protein were obtained after virtual screening by AutoDock Vina1.2.0 software using the above binding pocket as the docking site.

### 2.13 H&E staining

Hematoxylin and eosin (H&E) staining was performed to evaluate tissue morphology. Briefly, tumor tissues were collected and fixed in 4% paraformaldehyde overnight at 4°C. The samples were then dehydrated through a graded series of ethanol (70%, 85%, 95%, and 100%) and embedded in paraffin. Thin tissue sections (5 μm) were cut and mounted onto glass slides. The sections were deparaffinized and rehydrated before staining with hematoxylin for 5 minutes and eosin for 3 minutes. After washing and dehydration, the slides were mounted with neutral balsam and observed under a light microscope.

### 2.14 Tumor dissociation

The tumor dissociation procedure was performed to obtain a single-cell suspension for subsequent flow cytometry analysis. Mouse tumor tissues were first excised and placed into a culture dish containing PBS (Procell). The tissues were minced using scissors and transferred into a new culture dish containing enzymatic digestion solution [Collagenase IV 2 mg/mL, DNase I 0.1 mg/mL, RPMI-1640 medium (Procell)] and incubated at 37°C for 1 hour, with gentle shaking every 15 minutes to facilitate complete digestion. Collagenase IV (Thermo Fisher Scientific) assisted in the breakdown of the extracellular matrix, while DNase I (Thermo Fisher Scientific) aided in the removal of nuclear debris. After digestion, the tissue was filtered through a 70 μm cell strainer to eliminate undigested tissue chunks and impurities. The filtered cell suspension was centrifuged (300×g for 5 minutes) to collect the cell pellet. The pellet was resuspended in red blood cell lysis solution (Biyuntian Biotechnology) and incubated at room temperature for 5 minutes with gentle shaking to ensure uniform lysis. The suspension was washed twice with PBS (Procell) to remove any residual lysis solution and cell debris. Finally, the cell pellet was resuspended in PBS, resulting in a single-cell suspension suitable for flow cytometry analysis.

### 2.15 RNA interference

Small interfering RNA (siRNA) oligonucleotides targeting *NANOS1* were provided by Xianghong Biotech Co., Ltd. (Beijing, China) (**Supplementary Table S1**). Each siRNA was transfected using Lipofectamine 3000 (Invitrogen). The specific sequences of the target gene are provided in the supplementary information.

### 2.16 Quantitative real-time PCR analysis

Total RNA was extracted using Trizol reagent (Invitrogen) and reverse transcribed using the PrimeScript™ RT reagent kit (Takara). Quantitative real-time PCR (qRT-PCR) was performed in triplicate using TB Green Premix Ex Taq (Takara). The primer sequences used for qRT-PCR of the target genes are listed in **Supplementary Table S1**.

### 2.17 Flow cytometry

Flow cytometry was used to analyze the immune cell populations within the tumor microenvironment. The single-cell suspension was aliquoted into two tubes for different fluorescent antibody labeling. One tube was labeled with F4/80, CD11b, CD45.2 antibodies, while the other tube was labeled with CD3e, CD4, CD8a, CD279 (PD-1), CD223 (LAG-3), and CD366 (TIM3) antibodies, all obtained from Thermo Fisher Scientific. The cells were incubated on ice for 30 minutes, allowing surface antigens on the cell membranes to bind to the respective fluorescently labeled antibodies. Following incubation, the cells were washed twice with PBS (Procell) to remove unbound antibodies. The cells were then fixed with 4% paraformaldehyde for 15 minutes, followed by two PBS washes. After all staining and fixation steps, the cells were washed again and resuspended in PBS for flow cytometry analysis. Antibodies were purchased from Thermo Fisher Scientific, and their specific details are provided in **Supplementary Table S2**.

Flow cytometry data were acquired using a BD FACSCalibur flow cytometer. Initial gating was performed using forward scatter (FSC) and side scatter (SSC) plots. Immune phenotype analysis was conducted using multiple fluorescence channels. The flow cytometry data were analyzed with FlowJo software, and FSC and SSC gating were used to select the appropriate cell populations. Immune phenotype analysis was based on distinct fluorescence markers. The gating strategy is outlined in **Supplementary Figure S1**.

### 2.18 Western blot assay

Cells were treated with Dig and AA for 48 hours, then lysed on ice using RIPA buffer (Solarbio) containing 1% PMSF. Protein concentration was determined using the Pierce BCA Protein Assay Kit (Thermo Fisher Scientific). Equal amounts of protein (20 µg) were separated by 10% SDS-PAGE (Vazyme), transferred to a PVDF membrane (Millipore), and blocked with 5% nonfat milk at room temperature. The membrane was incubated overnight with primary antibody at 4°C, washed with TBST, and incubated with secondary antibody at room temperature. Protein signals were detected using a high-sensitivity ECL chemiluminescent detection kit (Proteintech) on the BG-gdsAUTO730. Anti-NOS-1 was purchased from abcam (working concentration: 2 µg/mL), Beta Actin Polyclonal antibody from ProteinTech Group, Inc. (1:2000 dilution), and Multi-rAb HRP-Goat Anti-Rabbit Recombinant Secondary Antibody (H+L) from ProteinTech Group, Inc. (1:5000 dilution).

### 2.19 Statistical analysis

Data were collected from independent experiments and expressed as the mean ± SEM. All graphs and analyses were generated using GraphPad Prism software. The significance among multiple (three or more) groups was compared using one-way ANOVA analysis, and differences between the different groups were analyzed by a Student’s *t*-test.

## 3 Results

### 3.1 Inhibition of growth, migration and invasion of TNBC by TOE

**Figure 1:**
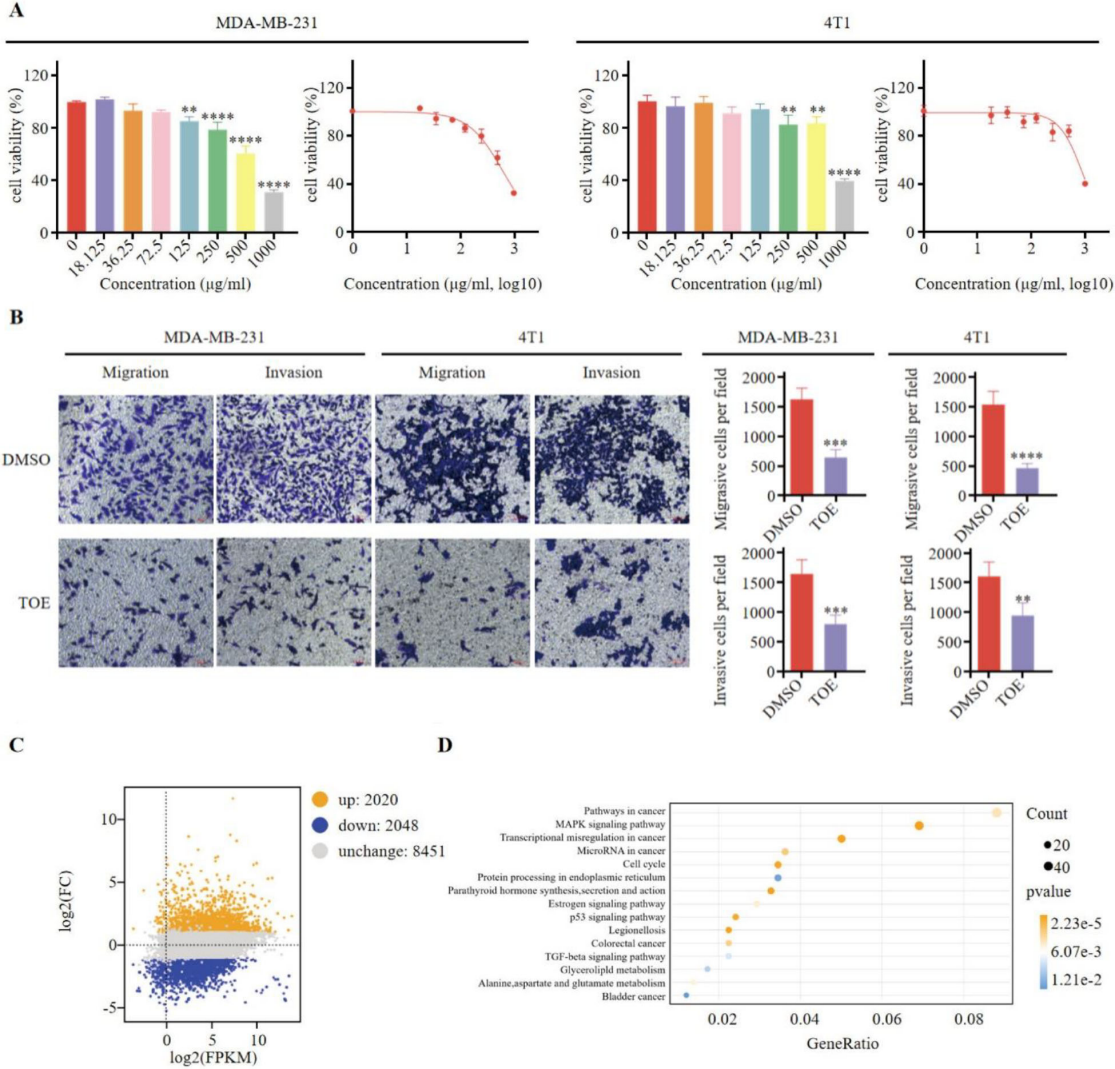
TOE suppresses malignant phenotype of triple negative breast cancer cells. **(A)** The effect of TOE on cell proliferation in MDA-MB-231 and 4T1 cells. Cells were treated with varying concentrations of TOE for 24 hours, and cell proliferation was assessed using the CCK-8 assay. Data are expressed as the mean ± SEM (n = 3). Statistical significances were calculated via Student’s t-test. **p < 0.01 and ***p < 0.001 and ****p < 0.0001. **(B)** Transwell migration and invasion assay of MDA-MB-231 and 4T1 cells after treatment with TOE for 24 hours. Representative images of the migrated and invaded cells from randomly selected fields of Transwell inserts are shown on the left, while quantitative data for cell numbers are presented on the right. Cell numbers were calculated and expressed as the mean ± SEM of three independent experiments. Statistical significance was determined by t-test, with **p < 0.01 and ***p < 0.001 and ****p < 0.0001 indicating significant differences between TOE-treated and DMSO-treated cells. Scale bar = 100 μm. **(C)** MA plot of DGEs in MDA-MB-231 treated with TOE. **(D)** Enrichment and scatter map of KEGG pathway of DGEs.

As shown in **Figure 1A**, TOE significantly inhibited the growth of MDA-MB-231 and 4T1 cells, with the inhibitory effect increasing in a concentration-dependent manner. Additionally, TOE reduced the migration and invasion abilities of these cells (**Figure 1B**). MDA-MB-231 cells were treated with an IC50 dose of TOE (635.4 μg/ml), and RNA sequencing (RNA-seq) was performed to assess their transcriptomes. A total of 8451 differentially expressed genes (DEGs) were identified following 24 hours of treatment with TOE, with 2,020 genes upregulated and 2,048 genes downregulated (**Figure 1C**). The top 600 upregulated and downregulated genes were selected for KEGG pathway enrichment analysis based on the |log2(FC)| values, and the top 20 pathways with the lowest p-values are shown in **Figure 1D**. The five most significantly enriched pathways included pathways in cancer, the MAPK signaling pathway, transcriptional misregulation in cancer, microRNA in cancer, and the cell cycle.

### 3.2 Correlation of NANOS1 protein with prognosis and immune cell infiltration in TNBC

**Figure 2:**
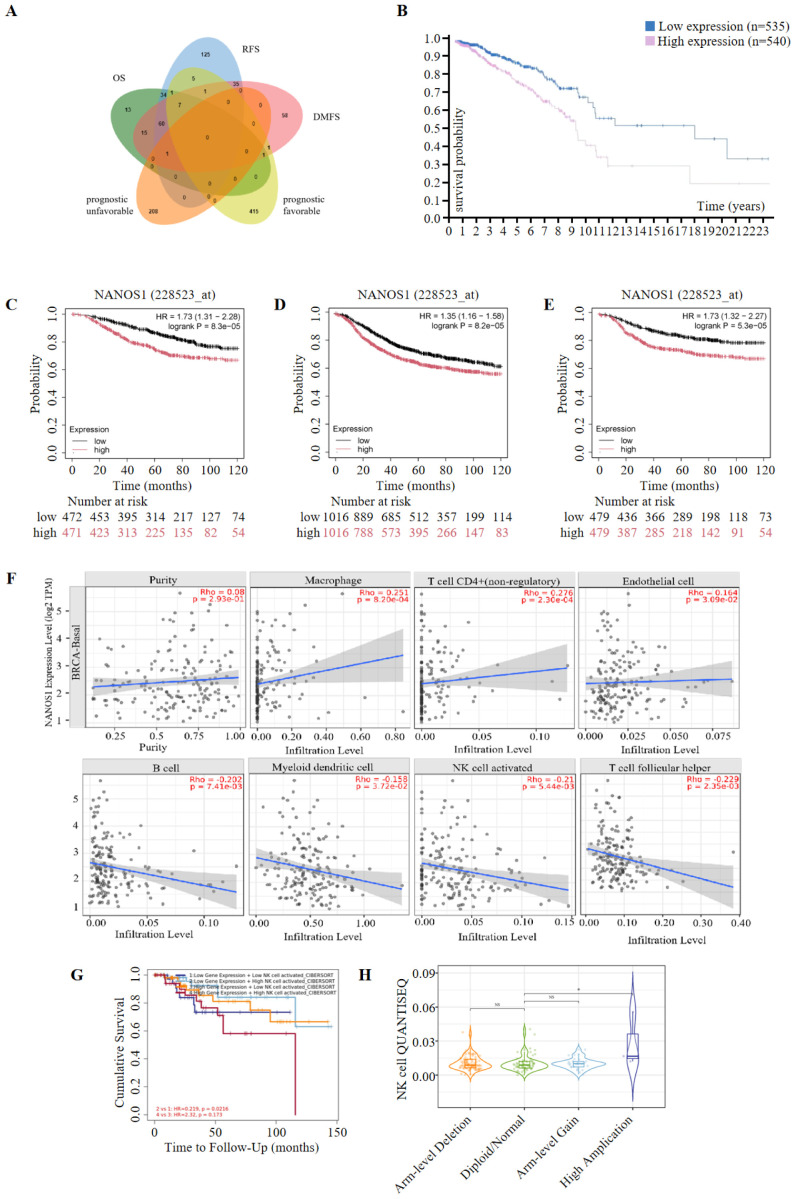
Prognostic significance of *NANOS1* and its association with immune infiltration in breast cancer. **(A)** Venn diagram of prognostic genes. Genes were selected based on RNA-Seq data (top 600 upregulated and 600 downregulated genes). Prognostic evaluation was conducted using the Kaplan-Meier Plotter tool (https://kmplot.com/analysis/), which integrates data from GEO, EGA, and TCGA. Statistical significance was determined using the log-rank test (p < 0.05). Genes were further validated using protein expression data from the Human Protein Atlas (HPA, https://www.proteinatlas.org/). The Venn diagram was created using the EVenn online tool (http://www.ehbio.com/test/venn/#/). **(B)** Survival curves in breast cancer at the protein level (n=1075) (www.proteinatlas.org). Data were obtained from the Human Protein Atlas (www.proteinatlas.org), and Kaplan-Meier survival curves were constructed based on protein expression levels of NANOS1. The statistical significance of survival curves was calculated using the log-rank test. **(C-E)** Survival curves of overall survival (OS), relapse-free survival (RFS), and distant metastasis-free survival (DMFS) in breast cancer at the mRNA level. Data were obtained from the Kaplan-Meier Plotter database (n = 943 for OS, n = 2032 for RFS, n = 958 for DMFS) (www.kmplot.com). Kaplan-Meier survival analysis was performed to evaluate the prognostic significance of NANOS1 expression at the mRNA level, with log-rank p-values shown for each curve. **(F)** Correlation between *NANOS1* expression and the level of immune infiltration. **(G)** The correlation between clinical outcomes and NK cell infiltration with NANOS1 expression in the BRCA-BASAL. The clinical relevance of tumor immune subtypes was explored using TIMER2.0 (http://timer.cistrome.org/), which shows NK cell immune infiltration levels and Kaplan-Meier survival curves based on NANOS1 expression. NK cell infiltration levels were categorized as low or high. The hazard ratios and p-values from the Cox proportional hazards model, along with the log-rank p-value from the Kaplan-Meier analysis, are displayed on the survival curves. Data source: TIMER2.0 database, using gene expression profiles and clinical data. **(H)** Violin plot showing NK cell infiltration levels in relation to sCNA amplifications of NANOS1 in BRCA-Basal. The immune infiltration distribution was analyzed using TIMER2.0 (http://timer.cistrome.org/) based on the sCNA (somatic copy number alteration) status of the gene NANOS1. The sCNA data were obtained from gene-level copy number segmentation, including “arm-level deletion,” “diploid/normal,” “arm-level gain,” and “high amplification” categories defined by GISTIC2.0. NK cell infiltration levels were assessed using the QUANTISEQ method. Significant differences in NK cell infiltration were observed between the “high amplification” and “normal” samples (p = 0.032).

Kaplan-Meier survival analysis was performed to assess the prognostic significance of the top 600 upregulated and downregulated differentially expressed genes (DEGs). Genes positively correlated with survival were identified, focusing on the low expression of downregulated genes and the high expression of upregulated genes. A total of 132, 269, and 179 genes were found to be positively associated with overall survival (OS), relapse-free survival (RFS), and distant metastasis-free survival (DMFS), respectively. Subsequently, 640 prognostic genes were selected based on TCGA data and antibody-based protein data. Of these, 209 genes were associated with an unfavorable prognosis, while 431 genes were linked to a favorable prognosis for breast cancer at the protein level. Ultimately, 8 genes were identified as being associated with prognosis at both the transcript and protein levels (**Figure 2A**, **Table 1**). Among these, NANOS1, a less studied and the only downregulated gene, was chosen for further analysis. The prognostic value of NANOS1 protein expression in breast cancer was evaluated using the online tool available at www.proteinatlas.org, and the results demonstrated that lower expression of NANOS1 was associated with better prognosis (p < 0.001) (**Figure 2B**). At the mRNA level, reduced NANOS1 expression was also positively correlated with OS (p < 0.001), RFS (p < 0.001), and DMFS (p < 0.001) in breast cancer patients (**Figures 2C–E**).

**Table 1:** DGEs associated with prognosis in breast cancer.

ID	Symbol	log2FC	FDR	Regulated	Prognostics
ENSG00000188613	NANOS1	-2.099116259	5.3654288157e-26	down	unfavorable
ENSG00000159388	BTG2	3.827077677	1.4953679154e-91	up	favorable
ENSG00000121104	FAM117A	2.05076956	3.0029034189e-31	up	favorable
ENSG00000152804	HHEX	3.401245139	1.0481947934e-253	up	favorable
ENSG00000164128	NPY1R	3.055241553	1.1667617660e-09	up	favorable
ENSG00000181788	SIAH2	2.694286398	8.4419828317e-207	up	favorable
ENSG00000163659	TIPARP	2.650981515	0	up	favorable
ENSG00000160908	ZNF394	2.047360395	2.2696551915e-113	up	favorable

The correlation between NANOS1 expression and immune cell infiltration levels in BRCA-BASAL was assessed, given the association of immune infiltration with cancer development and treatment outcomes. Based on deconvolutional procedure, *NANOS1* expression was positively associated with Macrophage (*P*=8.20e-04), CD4+ T cell (non-regulatory) (*P*=2.30e-04) and Endothelial cell (*P*=3.09e-02), meanwhile, *NANOS1* expression was negatively associated with B cell (*P*=7.14e-03),Myeloid dendritic cell (*P*=3.72e-02), activated NK cell (*P*=5.44e-03), and T cell follicular helper (*P*=2.35e-03) (**Figure 2F**). Among these immune cells, NK cell infiltration was associated with a reduced risk of TNBC, and survival analysis revealed that high NK cell infiltration correlated with better prognosis in patients with low NANOS1 expression (*P*=0.0216) (**Figure 2G**). Furthermore, the level of NK cell infiltration in the somatic copy number amplifications (sCNA-Amplifications) state of *NANOS1* was demonstrated by violin plots. As shown in **Figure 2H**, a significant difference in NK cell infiltration was observed between high amplification samples and normal samples (*p*=0.032).

### 3.3 FDA-approved drugs virtual screening to NANOS1 proteins

**Figure 3:**
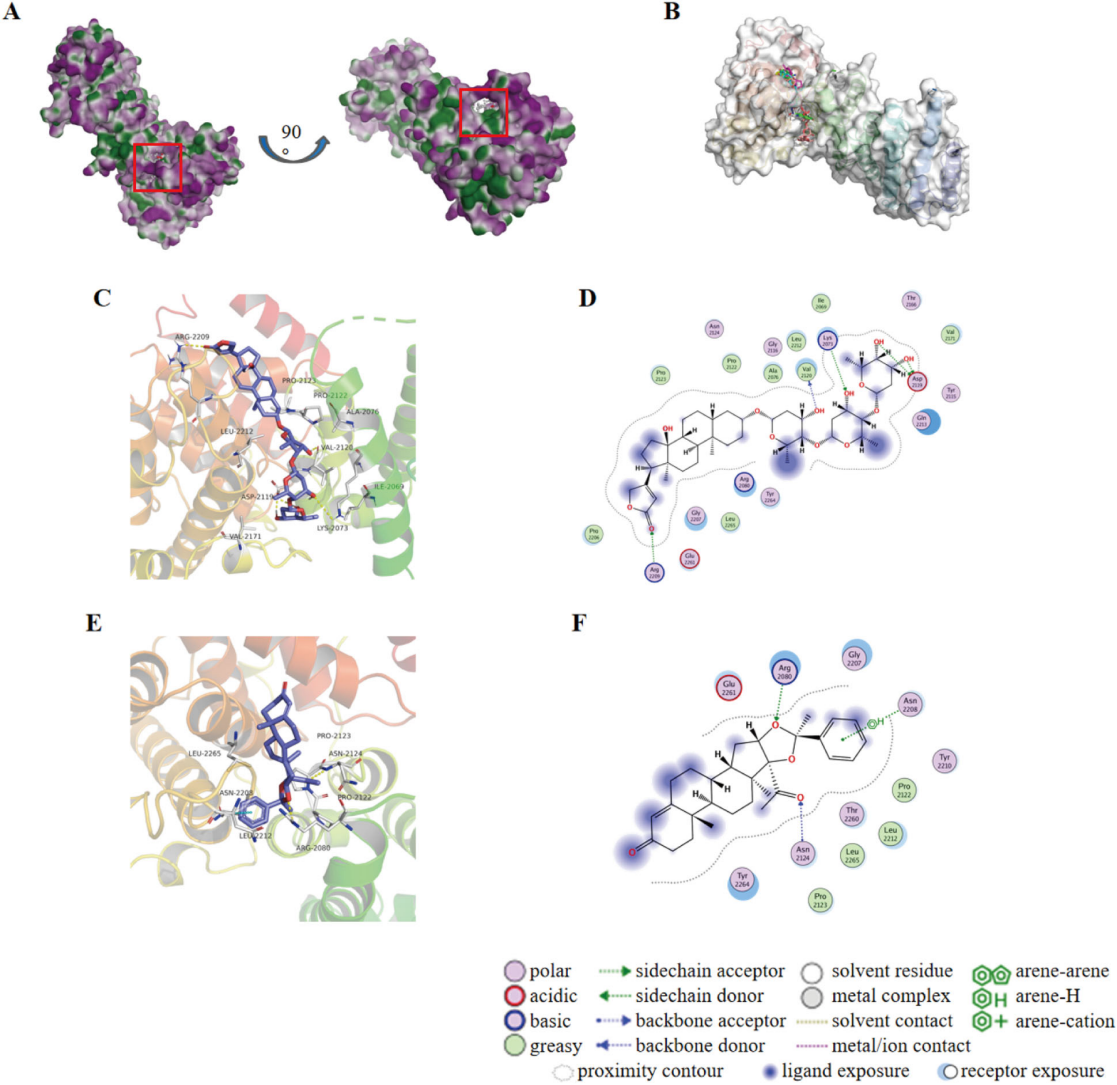
Molecular docking results of Dig and AA against NANOS1 protein. **(A)** Target binding pockets on the NANOS1 protein. The SimteMap tool analyzes the surface of the NANOS1 protein, highlighting potential binding pockets that could serve as targets for therapeutic intervention. **(B)** An overview of the binding modes of the six top-ranked compounds with distinct scaffolds. The figure presents the interaction patterns of each compound with the NANOS1 protein. The binding modes are visually represented in 3D molecular models, illustrating the orientation and interactions with the protein’s active site. **(C)** 3D diagram of the interaction between Dig and the NANOS1 protein. Dig forms five hydrogen bonds, primarily involving hydroxyl groups as hydrogen bond donors and acceptors, with one hydrogen bond formed between the carbonyl group of the terminal cyclic ether and Arg2209. There are no π-stacking or salt bridge interactions. Hydrophobic interactions are also observed due to the hydrophobic backbone of Dig. The interacting amino acids are shown as stick models in gray, with O atoms in red, N atoms in blue, and C atoms in gray. Yellow and cyan dotted lines indicate intermolecular hydrogen bonds and π-stacking interactions. **(D)** 2D diagrams of the docked structure of Dig in the active domain of NANOS1. **(E)** 3D diagram of the interaction between AA and the NANOS1 protein. AA forms two hydrogen bonds, one H-π stacking interaction, and several hydrophobic interactions with the protein. The hydrogen bonds are formed between Arg2080 and the oxygen on the furan ring, and between the oxygen on the carbonyl group and Asn2124. **(F)** 2D diagrams of the docked structure of AA in the active domain of NANOS1.

Typically, targetable proteins need to have a typical binding pocket, so the first step in this study was to use the SimteMap tool to analyze the targetable binding pocket on the surface of the NANOS1 protein. By calculation, SiteMap found a large potential ligand binding pocket consisting of the following amino acids: ALA2076, PRO2077, ARG2080, CYS2118, ASP2119, VAL2120, ILE2121, PRO2122, PRO2123, ASN2124, ARG2129, GLY2207, ASN2208, ARG2209, TYR2210, ASN2211, LEU2212, GLN2213, ASN2216, GLU2261, LEU2265, ASN2268, ALA2269, ASN2272, ARG2311. This indicates the presence of a targetable binding pocket for NANOS1 (**Figures 3A, B**). To explore potential interactions between approved drugs and the NANOS1 protein, we specifically selected drugs from the ZINC database for virtual docking simulations. The ZINC database was chosen due to its extensive collection of commercially available, drug-like molecules, as well as its provision of detailed compound information, including structural data and molecular properties, which are essential for accurate molecular docking. This approach allowed us to identify candidate drugs that may bind effectively to NANOS1. Next, the binding affinity of FDA-approved drugs was predicted using AutoDock Vina. A total of 3177 drug molecules were ranked based on their docking scores, from highest to lowest. The results indicated that Dig and AA both achieved the highest scores, with a docking score of -9.9 kcal/mol for each drug (a larger absolute value indicates stronger binding affinity) (**Table 2**). The binding modes were then visualized. **Figures 3C, D** show the interaction between Dig and the NANOS1 protein, where Dig forms five hydrogen bonds with the protein. This interaction involves hydroxyl groups as hydrogen bond donors and acceptors, and a hydrogen bond is formed between the carbonyl group of the terminal cyclic ether and Arg2209. No π-stacking or salt bridge interactions were observed. Additionally, Dig exhibits significant hydrophobic interactions with the protein due to its hydrophobic backbone. **Figures 3E, F** illustrate the interaction between AA and the NANOS1 protein. AA forms two hydrogen bonds, one H-π stacking interaction, and hydrophobic interactions with the protein. The hydrogen bonds occur between the side chain of Arg2080 and the oxygen atom on the furan ring, as well as between the oxygen atom on the carbonyl group and the amide backbone of Asn2124. The π-stacking interaction occurs between the charge center of Asn2208 and the phenyl ring at the terminal end of AA. Additionally, AA interacts with several hydrophobic amino acids such as Pro2122, Pro2123, Leu2265, and Leu2212.

**Table 2:** Docking scores and drug information of Top6 molecules.

ZIN No.	Docking score	Drugs
ZINC08101076	-9.9	Digoxin
ZINC03830650	-9.9	Algestone acetophenide
ZINC03831193	-9.7	Nandrolone phenylpropionate (NPP)
ZINC11592964	-9.6	Daunorubicin
ZINC01482077	-9.5	Gliquidone
ZINC03830767	-9.5	Estradiol Benzoate

### 3.4 Dig and AA inhibit tumor growth in TNBC mouse models and suppress malignant cellular phenotypes

**Figure 4:**
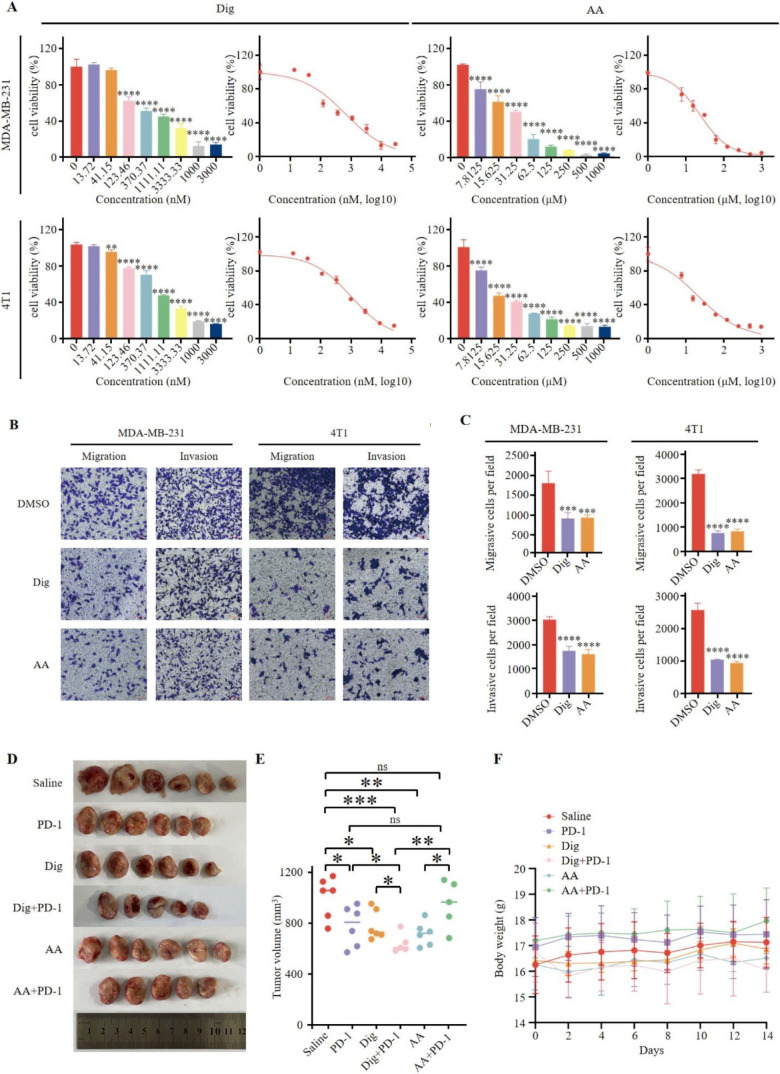
Dig and AA inhibited tumor growth in breast cancer mouse models. **(A)** Inhibition of growth by Dig and AA in MDA-MB-231 and 4T1 cells for 24 h. MDA-MB-231 and 4T1 cells were treated with Dig and AA (at various concentration) for 24 hours, and cell proliferation was assessed using the CCK-8 assay. Data are presented as the mean ± SEM from three independent experiments (n = 3). Statistical significance was determined using unpaired *t*-tests, with *p < 0.05, **p < 0.01, ***p < 0.001, and ****p < 0.0001 indicating significant differences compared to the DMSO control. **(B)** Transwell migration and invasion assay of MDA-MB-231 and 4T1 cells with Dig and AA treatment for 24 h. Representative images from randomly selected fields of transwell inserts, and Scalebar = 100 μm. **(C)** Quantitative data from the Transwell migration and invasion assays. Cell numbers were calculated and are expressed as the mean ± SEM of three independent experiments. * *p* < 0.05,** *p* < 0.01, *** *p* < 0.001 and ****p < 0.0001, as determined by unpaired *t*-tests. **(D)** Diagrammatic representation of tumor volume measurement. The diagram illustrates the measurement method, including caliper-based measurements of length and width used to calculate tumor volume (Volume = 1/2 × length × width^2). **(E)** Tumor sizes at day 14. **(F)** The body weight changes of mice in the period of 14 days after different treatments. The body weight of mice was monitored every 2 days after Dig and AA treatment. Data are expressed as the mean ± SEM. No significant changes in body weight were observed, suggesting that the treatments did not cause overt toxicity in mice.

Dig and AA were selected for *in vitro* validation. CCK-8 assays showed that both drugs significantly reduced cell viability, inhibited proliferation, and slowed growth (**Figure 4A**). The 24-hour IC50 values for Dig were 0.6806 µM and 1.162 µM for MDA-MB-231 and 4T1 cells, respectively, and for AA, 23.25 µM and 21.63 µM. Transwell assays demonstrated that Dig and AA significantly reduced the migration and invasion of MDA-MB-231 and 4T1 cells (**Figures 4B, C**). To assess the potential of Dig and AA in inhibiting TNBC tumor growth *in vivo*, a 4T1 mouse tumor model was established. Mice were randomly divided into six groups, receiving different treatments: PD-1 inhibitor monotherapy (5 mg/kg), Dig (5 mg/kg), AA (10 mg/kg), Dig + PD-1 inhibitor combination, AA + PD-1 inhibitor combination, and a saline control. Both Dig and AA significantly inhibited tumor growth compared to the control group (**Figure 4D**). Moreover, the combination of Dig and PD-1 inhibitor showed superior tumor suppression compared to PD-1 inhibitor alone, highlighting the potential of this combination as a therapeutic strategy for TNBC (**Figure 4E**). No significant weight changes were observed in the combination treatment groups (**Figure 4F**).

### 3.5 Combination therapy of Dig with PD-1 inhibitor enhances antitumor immune response

**Figure 5:**
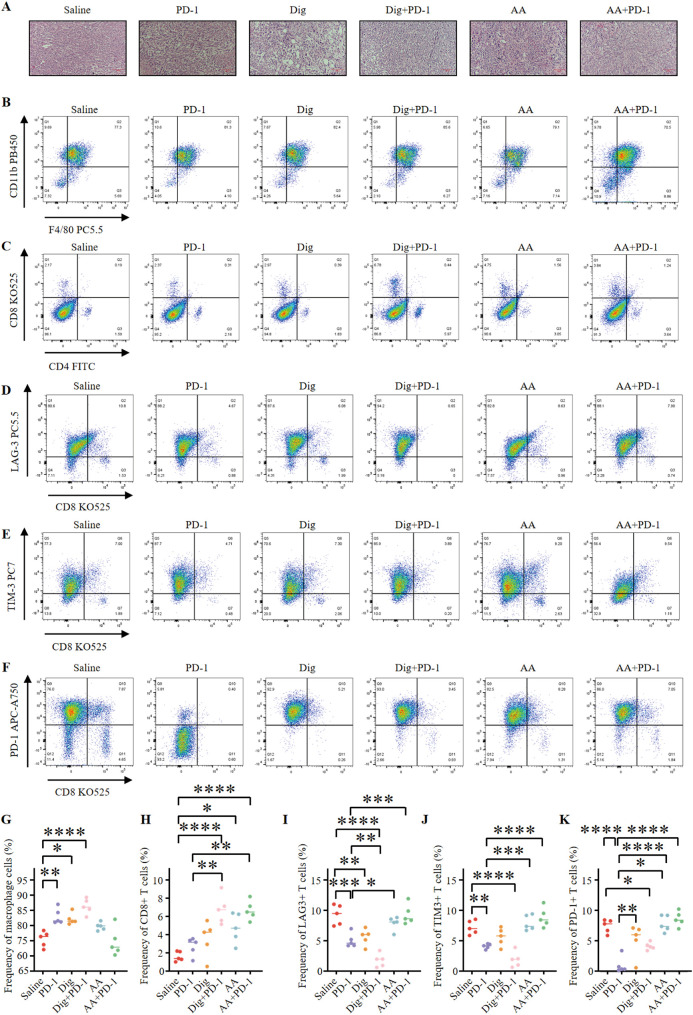
Dig combined with PD-1 immunotherapy can promote immune stimulation of *in situ* 4T1 breast tumors. **(A)** HE staining of mouse tumor tissues (scale bar = 100 μm). **(B)** Representative flow cytometry plots showing tumor-associated macrophages (TAMs) (CD45.2+, CD11b+, F4/80+) were obtained after different treatments. **(C)** Representative flow cytometry plots showing tumor immune cells after different treatments, including CTLs (CD45+, CD3+, CD8+) and Th cells (CD45+, CD3+, CD4+). **(D)** Representative flow cytometry plots demonstrating tumor-infiltrating LAG-3+ exhausted T cells (CD3+, CD8+, LAG-3+) after different treatments. **(E)** Representative flow cytometry plots demonstrating tumor-infiltrating TIM-3+ exhausted T cells (CD3+, CD8+, TIM-3+) after different treatments. **(F)** Representative flow cytometry plots demonstrating tumor-infiltrating PD-1+ exhausted T cells (CD3+, CD8+, PD-1+) after different treatments. **(G)** The levels of TAMs were quantified through flow cytometry analysis (n = 5). Statistical analysis was performed using Tukey’s multiple comparison test. The following adjusted p-values were obtained for each comparison: Saline vs PD-1: 0.0033; Saline vs Dig: 0.0106; Saline vs Dig+PD-1: <0.0001; Saline vs AA: 0.2039; Saline vs AA+PD-1: 0.9935; PD-1 vs Dig: 0.9961; PD-1 vs Dig+PD-1: 0.4395; PD-1 vs AA: 0.4330; PD-1 vs AA+PD-1: 0.0009; Dig vs Dig+PD-1: 0.2080; Dig vs AA: 0.7273; Dig vs AA+PD-1: 0.0028; Dig+PD-1 vs AA: 0.0109; Dig+PD-1 vs AA+PD-1: <0.0001; AA vs AA+PD-1: 0.0715. **(H)** The levels of CTLs were quantified by flow cytometry analysis (n = 5). The following adjusted p-values were obtained for each comparison: Saline vs PD-1: 0.7941; Saline vs Dig: 0.2550; Saline vs Dig+PD-1: <0.0001; Saline vs AA: 0.0161; Saline vs AA+PD-1: <0.0001; PD-1 vs Dig: 0.9237; PD-1 vs Dig+PD-1: 0.0013; PD-1 vs AA: 0.2253; PD-1 vs AA+PD-1: 0.0016; Dig vs Dig+PD-1: 0.0136; Dig vs AA: 0.7542; Dig vs AA+PD-1: 0.0164; Dig+PD-1 vs AA: 0.2253; Dig+PD-1 vs AA+PD-1: >0.9999; AA vs AA+PD-1: 0.2576. **(I)** Flow cytometry analysis quantified the levels of LAG-3+ exhausted T cells (n = 5). Statistical analysis was performed using Tukey’s multiple comparison test. Flow cytometry analysis quantified the levels of LAG-3+ exhausted T cells (n = 5). Statistical analysis was performed using Tukey’s multiple comparison test. The following adjusted p-values were obtained for each comparison: Saline vs PD-1: 0.0006; Saline vs Dig: 0.0030; Saline vs Dig+PD-1: <0.0001; Saline vs AA: 0.5950; Saline vs AA+PD-1: >0.9999; PD-1 vs Dig: 0.9841; PD-1 vs Dig+PD-1: 0.0094; PD-1 vs AA: 0.0282; PD-1 vs AA+PD-1: 0.0005; Dig vs Dig+PD-1: 0.0019; Dig vs AA: 0.1152; Dig vs AA+PD-1: 0.0027; Dig+PD-1 vs AA: <0.0001; Dig+PD-1 vs AA+PD-1: <0.0001; AA vs AA+PD-1: 0.5676. **(J)** Flow cytometry analysis quantified the levels of TIM-3+ exhausted T cells (n = 5). Statistical analysis was performed using Tukey’s multiple comparison test. The following adjusted p-values were obtained for each comparison: Saline vs PD-1: 0.0073; Saline vs Dig: 0.3784; Saline vs Dig+PD-1: <0.0001; Saline vs AA: 0.9296; Saline vs AA+PD-1: 0.3206; PD-1 vs Dig: 0.4016; PD-1 vs Dig+PD-1: 0.0959; PD-1 vs AA: 0.0007; PD-1 vs AA+PD-1: <0.0001; Dig vs Dig+PD-1: 0.0011; Dig vs AA: 0.0698; Dig vs AA+PD-1: 0.0050; Dig+PD-1 vs AA: <0.0001; Dig+PD-1 vs AA+PD-1: <0.0001; AA vs AA+PD-1: 0.8546. **(K)** Flow cytometry analysis quantified the levels of PD-1+ exhausted T cells (n = 5). Statistical analysis was performed using Tukey’s multiple comparison test. The following adjusted p-values were obtained for each comparison: Saline vs PD-1: <0.0001; Saline vs Dig: 0.2205; Saline vs Dig+PD-1: 0.0276; Saline vs AA: 0.9984; Saline vs AA+PD-1: 0.8260; PD-1 vs Dig: 0.0043; PD-1 vs Dig+PD-1: 0.0469; PD-1 vs AA: <0.0001; PD-1 vs AA+PD-1: <0.0001; Dig vs Dig+PD-1: 0.9030; Dig vs AA: 0.1042; Dig vs AA+PD-1: 0.0181; Dig+PD-1 vs AA: 0.0108; Dig+PD-1 vs AA+PD-1: 0.0015; AA vs AA+PD-1: 0.9632. Data are expressed as the mean ± SEM. *p < 0.05, **p < 0.01, ***p < 0.001 and ****p < 0.0001 vs. Saline.

Freshly excised tumor tissues were harvested; one portion was used for flow cytometry analysis to assess immune cell populations, while the remaining portion was processed for H&E staining to examine tissue morphology and immune cell infiltration (**Figure 5A**). To evaluate the potential of combination therapy in promoting tumor lymphocyte infiltration and modulating the immunosuppressive environment within 4T1 tumors, several parameters were assessed. The combination of Dig and PD-1 significantly increased the frequency of tumor-associated macrophages compared to the control group (**Figures 5B, G**). Evaluation of effector T cells revealed that the combination therapy of Dig and PD-1 resulted in a significant increase in the frequency of effector CD8+ T cells compared to the saline group, and this effect was superior to the PD-1 monotherapy group. AA enhances the proportion of CD8+ T cells. However, although the AA + PD-1 inhibitor combination showed a more pronounced increase in CD8+ T cells, the lack of statistical significance (adjusted p value = 0.2576) indicates that the effect of the AA and PD-1 inhibitor combination is less effective than that of the Dig and PD-1 inhibitor combination (**Figures 5C, H**). These findings indicate that the combination therapy involving Dig effectively enhances the immune response to immune checkpoint blockade (ICB) therapy.

This study also investigated the effects of Dig and AA, either as monotherapies or in combination with PD-1 inhibitors, on exhausted T cells. The results showed that Dig, either alone or in combination with PD-1 inhibitors, effectively reduced the frequency of LAG-3-expressing exhausted T cells (**Figures 5D, I**). The combination of Dig and PD-1 exhibited synergistic effects in reducing TIM-3 expression levels (**Figures 5E, J**). Dig monotherapy also effectively reduced the frequency of PD-1-positive exhausted T cells (**Figures 5F, K**). The analysis of exhausted T cells revealed a concerning trend in the AA + PD-1 inhibitor group, where the proportions of LAG-3+, TIM-3+, and PD-1+ exhausted T cells were higher than those in the AA alone group. The adjusted p-values of 0.5676, 0.8546, and 0.9632 indicate an increase in T cell exhaustion with the AA + PD-1 combination, which may account for the diminished efficacy observed with this combination. This suggests that while AA alone exhibits significant antitumor activity, its combination with PD-1 inhibitors may induce a state of T cell exhaustion, potentially limiting the overall therapeutic benefit. These findings provide strong experimental evidence for the development of novel tumor immunotherapy strategies. The “drug repurposing” approach has emerged as a new paradigm in antitumor drug discovery, offering the advantage of bypassing established toxicological and pharmacokinetic evaluations. Furthermore, this study demonstrates that modulating the tumor microenvironment, as evidenced by increased immune cell infiltration in the tumor, can enhance the efficacy of existing immunotherapies, significantly reducing development time and research costs.

### 3.6 Dig and AA inhibit tumor progression by regulating *NANOS1* to suppress TNF-α expression

**Figure 6:**
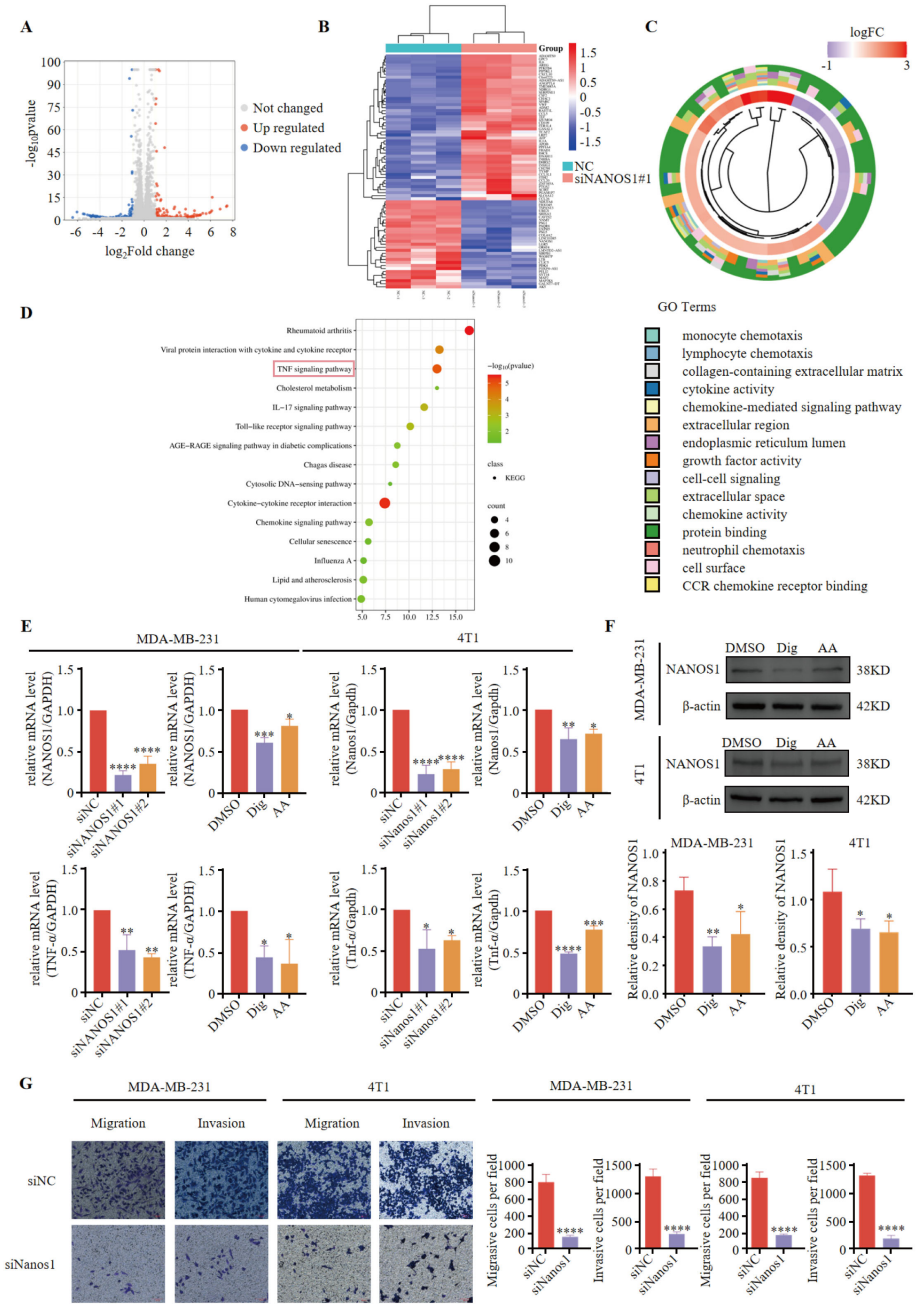
*NANOS1* Silencing Reduces TNF-α Expression and Cell Invasiveness. **(A)** Volcano plots of RNA-seq. Differential gene expression analysis was performed using RNA sequencing data from MDA-MB-231 cells with siNANOS1#1 silencing. The volcano plot shows the distribution of genes based on log-fold change versus the negative log-transformed p-value. Significant upregulated and downregulated genes are indicated with colored dots. Genes that meet the threshold of log-fold change (|logFC| > 2) and adjusted p-value < 0.05 are considered differentially expressed. **(B)** Heatmap of differential gene expression. **(C)** Chord diagram of GO enrichment results and related genes. **(D)** Top 15 KEGG pathways. The x-axis represents the gene ratio (*p <* 0.05), and the y-axis represents the enriched terms. **(E)** The knockdown efficiency of siRNA and the expression of NANOS1 and TNF-α following Dig and AA treatment were quantified by qPCR. MDA-MB-231 and 4T1 cells were transfected with siRNA targeting NANOS1, followed by treatment with Dig and AA. Quantitative PCR was performed to assess the expression levels of NANOS1 and TNF-α. The data are expressed as the mean ± SEM, with statistical significance calculated using one-way ANOVA. **(F)** Dig and AA decreased the expression of NANOS1 protein (n = 3). Data are expressed as the mean ± SEM. Statistical significances were calculated via one-way ANOVA, *p < 0.05, **p < 0.010, ***p < 0.001 and ****p < 0.0001 vs. DMSO. **(G)** Perforation migration and invasion assays of MDA-MB-231 and 4T1 cells after 24 h of siRNA treatment, scale bar = 100 mm. Cell numbers were calculated and are expressed as the mean ± SEM of three independent experiments. *p < 0.05, **p < 0.01, ***p < 0.001 and ****p < 0.0001, as determined by unpaired t-tests, were regarded as significant.

To investigate the role of NANOS1, we silenced its expression using siNANOS1#1 in MDA-MB-231. The silencing of NANOS1 was confirmed by quantitative PCR analysis. Differential gene expression analyses identified a notable number of genes exhibiting upregulation and downregulation (**Figure 6A**). Among these, IL6 was identified as the most significantly differential gene, with an adjusted p-value of 4.67E-191. A differential gene expression heatmap was generated to visualize these changes (**Figure 6B**). These 77 differentially expressed genes were then subjected to GO and KEGG pathway analyses to explore their biological functions and relevant pathways (**Figures 6C, D**). KEGG biological pathway analysis showed gene enrichment on pathways such as the TNF-signal pathway. In the triple-negative breast cancer cell lines MDA-MB-231 and 4T1, siRNA interference, Dig, and AA treatments effectively downregulated the expression of NANOS1 (**Figure 6E**). Furthermore, Dig and AA treatments reduced the protein expression of NANOS1 in TNBC cell lines (**Figure 6F**). We discuss the role of TNF-α in promoting tumor cell vascular adhesion and supporting angiogenesis. Previous studies have shown that TNF-α enhances tumor metastasis by upregulating the expression of adhesion molecules and stimulating the expression of angiogenesis factors (28–30). Our experimental results indicate that siRNA interference, Dig, and AA treatments all suppress TNF-α gene expression, which is consistent with previous findings. Transwell assays revealed that siRNA-mediated silencing of *NANOS1* expression significantly reduced the migration and invasion abilities of MDA-MB-231 and 4T1 cells (**Figure 6G**).

## 4 Discussion

TNBC is an aggressive, recurring and poorly prognosed malignancy. Compared to other breast cancer subtypes, the mortality rate within 5 years of diagnosis is approximately 40% (31) and is more prone to distant metastases to visceral and brain (32, 33). Since TNBC tumors lack ER, PR and HER2, it is not sensitive to either endocrine therapy or molecular targeted therapy, and chemotherapy remains the standard approach to treatment (34). However, drug resistance of tumor cells and side effects of chemotherapy greatly limit the application of chemotherapy in the treatment of TNBC. Therefore, there is still an urgent need for new drugs with high efficiency and low toxicity for the treatment of TNBC.

Recent studies have demonstrated that *Taraxacum officinale* exhibits promising effects in the treatment of TNBC, with a low risk of treatment-related adverse effects, increasing its appeal. Nassan (35) observed that administering TOE to breast cancer-bearing mice for 4 weeks reduced the serum marker CA15-3, which is commonly used to monitor breast cancer progression. This effect was linked to the inhibition of the PI3K/Akt pathway, a signaling cascade often abnormally activated in tumorigenesis, drug resistance, and cancer progression. Furthermore, TOE may regulate endoplasmic reticulum stress and apoptosis by activating the PERK/p-eIF2α/ATF4/CHOP signaling pathway, leading to suppressed TNBC cell growth (36). In our study, TOE inhibited the growth, migration, and invasion of two TNBC cell lines, suggesting its potential therapeutic value. To explore the targets of TOE, we performed RNA sequencing analysis of MDA-MB-231 cells, followed by KEGG pathway enrichment analysis of the top 600 up-regulated and 600 down-regulated genes. The top five significantly enriched pathways were: pathways in cancer, MAPK signaling pathway, transcriptional misregulation in cancer, microRNAs in cancer, and the cell cycle. Among them, several MAPK signaling pathways have been shown to be closely associated with TNBC (37, 38), while some microRNAs members also play roles in carcinogenesis, metastasis, diagnosis, treatment and prognosis of TNBC (39). Transcriptional misregulation of breast cancer-related genes can likewise promote tumor development (40). This result provides confidence for *Taraxacum officinale* to treat breast cancer. Among the 1,200 differentially expressed genes, NANOS1 is the only gene whose protein levels are associated with breast cancer prognosis and are downregulated. Interestingly, the biological function of NANOS1 remains unannotated, highlighting the importance of our investigation into this gene.

*NANOS1* is a member of the *NANOS* gene family which encodes a CCHC-type zinc finger protein (41). It has been shown to promote tumor cell migration, dissemination, and invasion by displacing linker proteins and disrupting E-cadherin–dependent cell-cell adhesion (42). In this study, we analyzed the expression of NANOS1 in breast cancer and found that lower NANOS1 expression in breast cancer patients was associated with a better prognosis. Given that immune infiltration levels are linked to cancer progression and treatment outcomes, we further explored the correlation between NANOS1 expression and immune infiltration. Specifically, NANOS1 expression was negatively correlated with activated NK cells, and survival analysis revealed that high NK cell infiltration was associated with a better prognosis when NANOS1 expression was low.

Additionally, we investigated whether any approved drugs could target *NANOS1* to suppress the malignant phenotype of TNBC cells. Among the top six ranked drugs, nandrolone phenylpropionate (43) and daunorubicin (44) have been previously shown to be effective in treating breast cancer, suggesting that *NANOS1* is a novel target for these drugs. Notably, digoxin, a commonly used medication for heart disease, was also identified as a potential candidate targeting *NANOS1*. Its efficacy in treating breast cancer requires further validation, which presents an opportunity to explore its novel therapeutic applications.

This study demonstrated that the combination of Dig and PD-1 inhibitors significantly suppresses the growth of triple-negative breast cancer (TNBC) in an allograft mouse model. The combined therapy not only enhanced tumor infiltration of macrophages and CD8+ T cells but also reduced the proportion of exhausted T cells in the tumor microenvironment (TME), indicating its potential as a novel immunotherapeutic strategy. Our findings highlight the potential of combining FDA-approved drugs with PD-1 inhibitors as a strategy to enhance the efficacy of immune checkpoint blockade (ICB) in TNBC. By leveraging the synergistic effects of FDA-approved drugs and modern immunotherapies, this study provides a new perspective for exploring effective cancer combination therapies.

Digoxin is a commonly used cardiac glycoside for the treatment of heart failure and atrial fibrillation, but its narrow therapeutic index significantly increases the risk of toxicity. The recommended therapeutic plasma concentration of digoxin is 0.5 to 2 ng/mL, and concentrations exceeding this range can lead to severe adverse effects, including gastrointestinal symptoms, central nervous system disturbances, and life-threatening arrhythmias (45). Renal function plays a crucial role in the elimination of digoxin, and impaired renal function can result in drug accumulation, further increasing the risk of toxicity (46). In this experiment, we assessed the effects of digoxin monotherapy and combination therapy on mouse body weight, kidney and liver indices, with no significant differences compared to the saline control group. These preliminary findings suggest the safety of the combination therapy with digoxin. Therefore, close monitoring of serum digoxin concentrations, renal function, and electrolyte levels in subjects receiving the drug is essential to prevent toxicity and enhance the clinical translational value of this combination therapy.

The limited response of TNBC to immune checkpoint blockade (ICB) remains poorly understood, with one critical factor being the lack of activated immune cells within the tumor microenvironment (TME). This deficiency renders many TNBC tumors “cold,” resulting in poor responses to immunotherapy. Previous studies have shown that targeting the JAK1/STAT3 pathway with Aurora kinase inhibitors can promote the expression of Th1 chemokines such as CXCL10 and CXCL11, facilitating the conversion of “cold” tumors to “hot” tumors and thereby improving the efficacy of ICB (47). Similarly, our findings suggest that Dig can remodel the TME by promoting immune cell recruitment and antitumor immune responses, supporting its potential role as an adjuvant to ICB therapy. While our *in vivo* results are promising, further studies are required to elucidate the precise molecular targets and mechanisms of these compounds. Future research should focus on optimizing their dosage and combinations to maximize therapeutic potential.

## Data Availability

The datasets used in this study have been uploaded to the NCBI database with the accession number PRJNA1209407. Further inquiries can be directed to the corresponding authors.
