# Correction: Targeting NANOS1 in triple-negative breast cancer: synergistic effects of digoxin and PD-1 inhibitors in modulating the tumor immune microenvironment

**DOI:** 10.3389/fonc.2025.1654169

**Published:** 2025-09-02

**Authors:** Tangyi Wang, Yadian Lei, Jingwei Sun, Li Wang, Yuxin Lin, Zhijing Wu, Shoude Zhang, Chengzhu Cao, Haiyan Wang

**Affiliations:** ^1^ Department of Basic Medical Sciences, Qinghai University Medical College, Xining, Qinghai, China; ^2^ Department of Medical Laboratory, Qinghai Provincial People’s Hospital, Xining, Qinghai, China; ^3^ State Key Laboratory of Plateau Ecology and Agriculture, Qinghai University, Xining, Qinghai, China; ^4^ Research Center for High Altitude Medicine, Qinghai University, Xining, Qinghai, China; ^5^ Key Laboratory of the Ministry of High Altitude Medicine, Qinghai University, Xining, Qinghai, China

**Keywords:** immune checkpoint blockade, triple-negative breast cancer, malignant phenotype, nanos1, PD-1 inhibitors, tumor microenvironments

There was a mistake in **Figure 1B** as published. The figure contains an error caused by an unintentional assembly mistake: while adjusting the layout to keep the spacing and formatting uniform, we duplicated a placeholder image that inadvertently overwrote the correct panel, resulting in incorrect image usage. The corrected **Figure 1** and its caption appear below.

**Figure 1:**
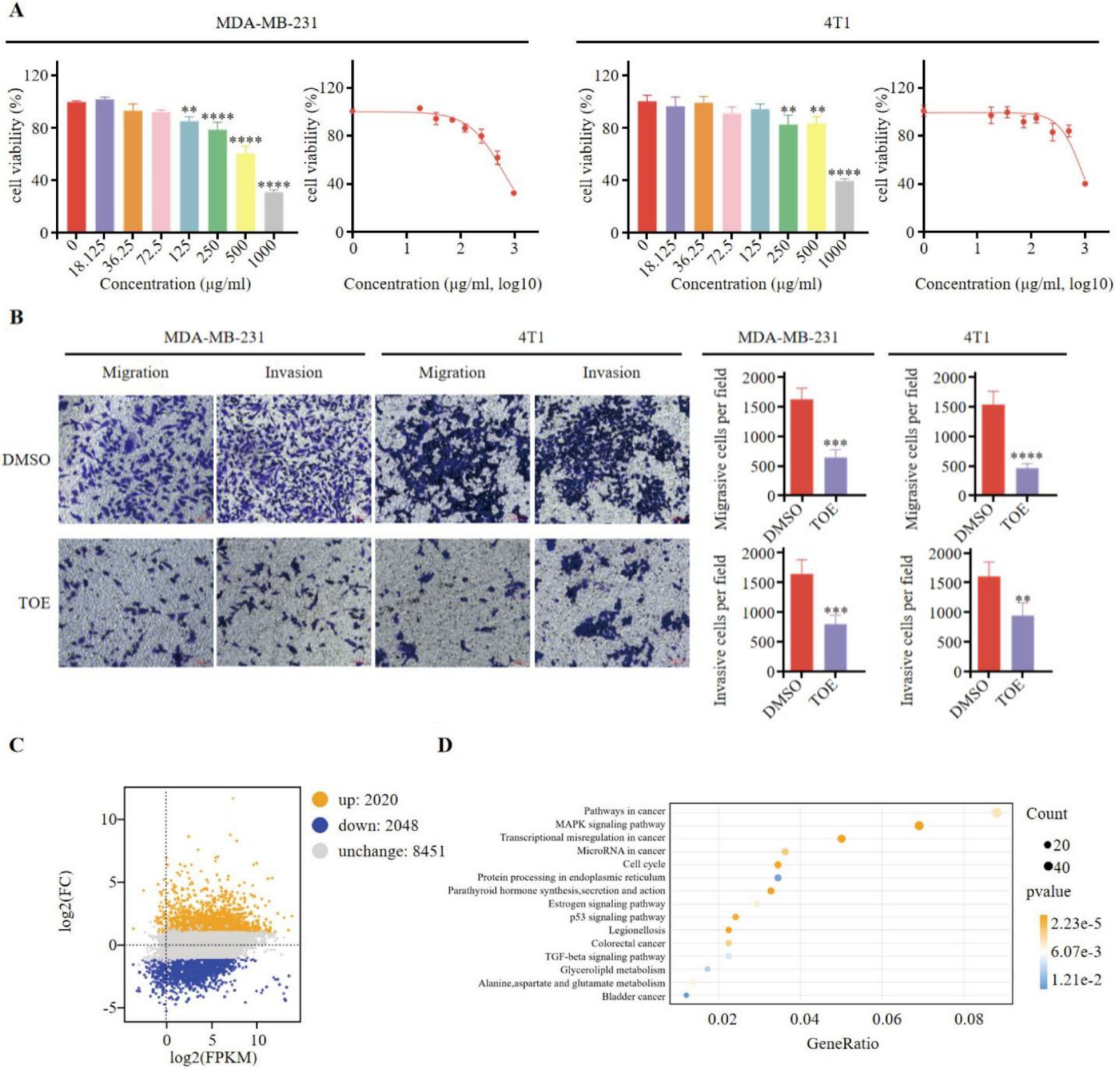
TOE suppresses malignant phenotype of triple negative breast cancer cells. **(A)** The effect of TOE on cell proliferation in MDA-MB-231 and 4T1 cells. Cells were treated with varying concentrations of TOE for 24 hours, and cell proliferation was assessed using the CCK-8 assay. Data are expressed as the mean ± SEM (n = 3). Statistical significances were calculated via Student’s t-test. **p < 0.01 and ***p < 0.001 and ****p < 0.0001. **(B)** Transwell migration and invasion assay of MDA-MB-231 and 4T1 cells after treatment with TOE for 24 hours. Representative images of the migrated and invaded cells from randomly selected fields of Transwell inserts are shown on the left, while quantitative data for cell numbers are presented on the right. Cell numbers were calculated and expressed as the mean ± SEM of three independent experiments. Statistical significance was determined by t-test, with **p < 0.01 and ***p < 0.001 and ****p < 0.0001 indicating significant differences between TOE-treated and DMSO-treated cells. Scale bar = 100 μm. **(C)** MA plot of DGEs in MDA-MB-231 treated with TOE. **(D)** Enrichment and scatter map of KEGG pathway of DGEs.

There was a mistake in **Figure 4B** as published. The figure contains an error due to an unintentional mistake during assembly, where the “4T1-Invasion-Dig” image was overwritten by the “MDA-MB-231-Invasion-AA” image, resulting in incorrect image usage. The corrected **Figure 4** and its caption appear below.

**Figure 4:**
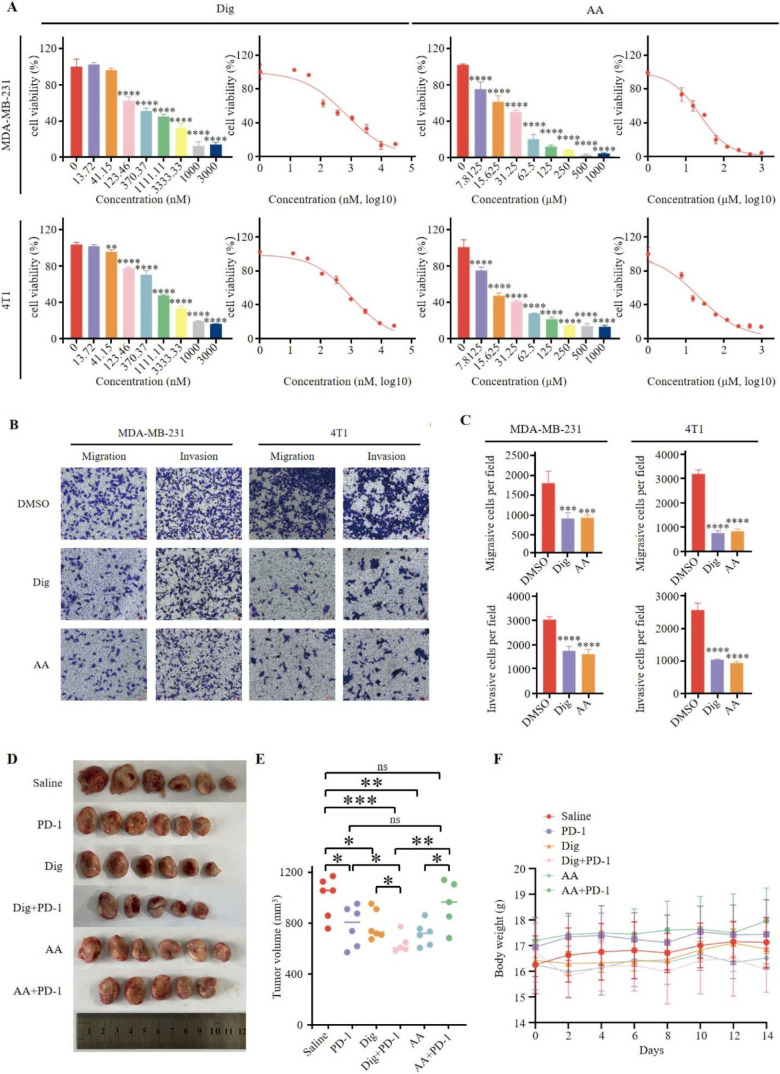
Dig and AA inhibited tumor growth in breast cancer mouse models. **(A)** Inhibition of growth by Dig and AA in MDA-MB-231 and 4T1 cells for 24 h. MDA-MB-231 and 4T1 cells were treated with Dig and AA (at various concentration) for 24 hours, and cell proliferation was assessed using the CCK-8 assay. Data are presented as the mean ± SEM from three independent experiments (n = 3). Statistical significance was determined using unpaired *t*-tests, with *p < 0.05, **p < 0.01, ***p < 0.001, and ****p < 0.0001 indicating significant differences compared to the DMSO control. **(B)** Transwell migration and invasion assay of MDA-MB-231 and 4T1 cells with Dig and AA treatment for 24 h. Representative images from randomly selected fields of transwell inserts, and Scalebar = 100 μm. **(C)** Quantitative data from the Transwell migration and invasion assays. Cell numbers were calculated and are expressed as the mean ± SEM of three independent experiments. * *p* < 0.05,** *p* < 0.01, *** *p* < 0.001 and ****p < 0.0001, as determined by unpaired *t*-tests. **(D)** Diagrammatic representation of tumor volume measurement. The diagram illustrates the measurement method, including caliper-based measurements of length and width used to calculate tumor volume (Volume = 1/2 × length × width^2). **(E)** Tumor sizes at day 14. **(F)** The body weight changes of mice in the period of 14 days after different treatments. The body weight of mice was monitored every 2 days after Dig and AA treatment. Data are expressed as the mean ± SEM. No significant changes in body weight were observed, suggesting that the treatments did not cause overt toxicity in mice.

The original version of this article has been updated.

