# Supplementary material for: Targeting NANOS1 in triple-negative breast cancer: synergistic effects of digoxin and PD-1 inhibitors in modulating the tumor immune microenvironment
Source: Front Oncol. 2025 Jan 24;14:1536406. doi: 10.3389/fonc.2024.1536406 (PMC11802438; doi:10.3389/fonc.2024.1536406)
Supplement: Supplementary file 1 [file DataSheet1.docx]

Supplementary Material

# Supplementary Tables

**Supplementary Table 1.** **Small interfering RNA sequences and qualitative PCR (qPCR) primer sequences for the genes interested.**

| Gene | species | | |  | Small interfering RNA sequence (5'→3') | |  |
| --- | --- | --- | --- | --- | --- | --- | --- |
| *NANOS1* | *Homo sapiens* | | | #1 | CUUCAGCUCCUGGAACGACUATT | |  |
|  |  | | |  | UAGUCGUUCCAGGAGCUGAAGTT | |  |
|  |  | | | #2 | CUACACCACCCAUAUCCUCAATT | |  |
|  |  | | |  | UUGAGGAUAUGGGUGGUGUAGTT | |  |
| *nanos1* | *Mus musculus* | | | #1 | GCGUUGGAACUGCGCGCACUATT | |  |
|  |  | | |  | UAGUGCGCGCAGUUCCAACGCTT | |  |
|  |  | | | #2 | GCCCAGCAAGAAGCUGCGCUATT | |  |
|  |  | | |  | UAGCGCAGCUUCUUGCUGGGCTT | |  |
| Gene |  | | |  | qPCR primer sequence (5'→3') | |  |
| *NANOS1* | |  | Forward | | | TCCTCCTCCTCGTCGTCCTG | |
|  | |  | Reverse | | | GTCGTCGTCCTCGTCGTAGTC | |
| *Nanos1* | |  | Forward | | | GCTTGCCACGCTCATCACC | |
|  | |  | Reverse | | | CTTCGCCGTCATCGTCTTCATC | |
| *TNF-α* | |  | Forward | | | CTCATCTACTCCCAGGTCCTCTTC | |
|  | |  | Reverse | | | CGATGCGGCTGATGGTGTG | |
| *Tnf-α* | |  | Forward | | | CACGCTCTTCTGTCTACTGAACTTC | |
|  | |  | Reverse | | | CTTGGTGGTTTGTGAGTGTGAGG | |
| *GAPDH* | |  | Forward | | | AGAAGGCTGGGCTCATTTG | |
|  | |  | Reverse | | | AGGGGCCATCCACATCTTTC | |
| *Gapdh* | |  | Forward | | | GTGGCAAAGTGGAGATTGTTG | |
|  | |  | Reverse | | | CGTTGAATTTGCCGTGAGTG | |

**Supplementary Table2: Antibodies used in flow cytometry**

| Antibodies | Catalog No. | dilution ratio |
| --- | --- | --- |
| F4/80 Monoclonal Antibody (BM8),PerCP-Cyanine5.5 | 45-4801-82 | 1: 5 |
| CD11b Monoclonal Antibody (M1/70), eFluor™450 | 48-0112-82 | 1: 1 |
| CD45.2 Monoclonal Antibody (104), FITC | 11-0454-82 | 1: 4 |
| CD86 (B7-2) Monoclonal Antibody (GL1), APC | 17-0862-81 | 1: 1 |
| CD206 (MMR) Monoclonal Antibody (MR6F3), PE | 12-2061-80 | 1: 2 |
| CD3e Monoclonal Antibody (145-2C11), eFluor™ 450 | 48-0031-80 | 1: 5 |
| CD4 Monoclonal Antibody (GK1.5), FITC | 11-0041-82 | 1: 2 |
| CD8a Monoclonal Antibody (53-6.7),Brilliant Violet™ 480 | 414-0081-80 | 1: 5 |
| CD279 (PD-1) Monoclonal Antibody (J43), APC-eFluorTM780 | 47-9985-82 | 1: 5 |
| CD223 (LAG-3) Monoclonal Antibody，PerCP-eFluor™ 710 | 46-2231-82 | 1: 5 |
| CD366 (TIM3) Monoclonal Antibody (RMT3-23), PE-Cyanine7 | 25-5870-82 | 1: 5 |

# Supplementary Figues


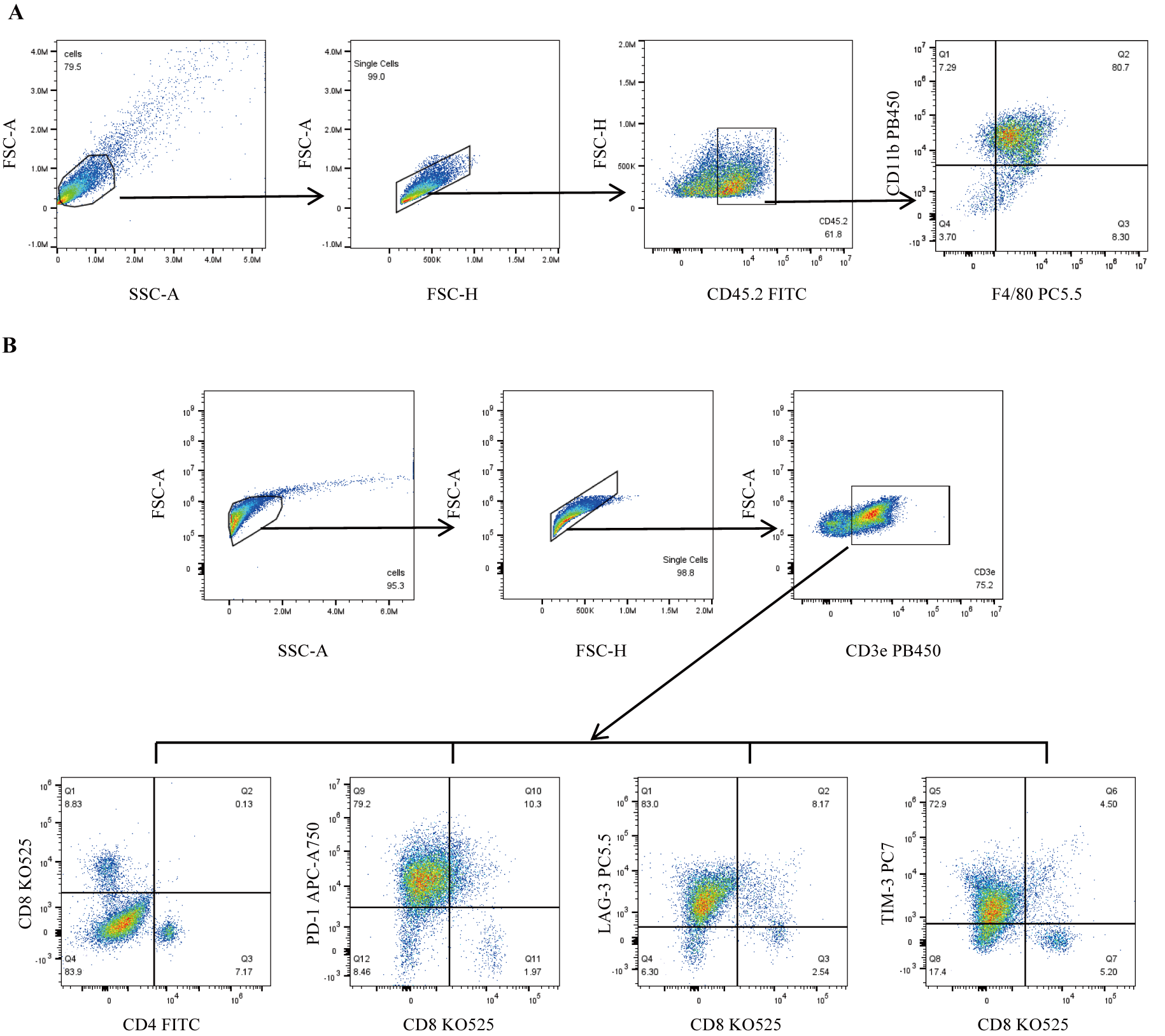


**Supplementary Figure 1.** **The gating strategy for flow cytometry of tumor-associated macrophages and tumor-infiltrating T cells.**

1. Flow cytometry gating strategies for tumor-associated macrophages.
2. Flow cytometry gating strategy for the expression of tumor-infiltrating CD4+ T cells, CD8+ T cells, LAG-3+ exhausted T cells, TIM-3+ exhausted T cells, and PD-1+ exhausted T cells.
